# The role of patient-provider communication: a qualitative study of patient attitudes regarding co-occurring depression and chronic diseases in Malawi

**DOI:** 10.1186/s12888-020-02657-2

**Published:** 2020-05-19

**Authors:** Christopher F. Akiba, Chifundo C. Zimba, Annie Thom, Maureen Matewere, Vivian Go, Brian Pence, Bradley N. Gaynes, Jones Masiye

**Affiliations:** 1 Department of Health Behavior, University of North Carolina at Chapel Hill, Gillings School of Global Public Health, 363 Rosenau Hall, CB# 7440, Chapel Hill, NC 27599 USA; 2 University of North Carolina Project Malawi, Tidziwe Center, 100 Mzimba Road, Private Bag A, /104 Lilongwe, Malawi; 3 Department of Epidemiology, University of North Carolina at Chapel Hill, Gillings School of Global Public Health, 2103C McGavran-Greenberg Hall, CB #7435, Chapel Hill, NC 27599 USA; 4 Department of Psychiatry, University of North Carolina at Chapel Hill, School of Medicine, 101 Manning Drive, Chapel Hill, NC 27514 USA; 5 Malawi Ministry of Health and Population, Non-communicable Diseases and Mental Health Clinical Services, P.O Box 30377, Lilongwe, 3 Malawi

**Keywords:** Integrated care, Depression, Non-communicable diseases, Low and middle-income countries, International health, Malawi, Patient-provider communication

## Abstract

**Background:**

Globally, depression is a leading cause of morbidity and mortality particularly in Low and Middle-Income Countries (LMICs). The burden of non-communicable diseases (NCDs) are also increasing in LMICs, the conditions frequently co-occur and exacerbate NCD outcomes. Depression interventions alone are not effective at improving NCD outcomes, resulting in wide-reaching calls for integrated services. Integrated services are in a nascent phase in LMICs in general and in Malawi in particular. This manuscript serves to clarify Malawian patients’ attitudes and perceptions towards depression integration into routine NCD services.

**Methods:**

Ten District Hospitals were selected for data collection and 2 participants were interviewed from each site (*N* = 20). An iterative approach to concept-driven coding was applied to facilitate the formation of similarities, differences, and connections between codes.

**Results:**

While patients infrequently described moderate depression as a medical condition, and held various attitudes regarding treatments, they agreed on the appropriateness of integrated services. Patients’ respect for their providers led them to support integration. Patients discussed how medical knowledge is highly regarded, revealing a power dynamic with their providers. Patients further acknowledged the importance of a provider’s communication in shaping a patient’s feelings about depression.

**Conclusions:**

Training and interventions that facilitate providers’ abilities to transfer their medical knowledge, use strategies to channel their power, and engage patients in a meaningful and collaborative relationship will be key to successfully integrating depression treatment into Malawian NCD clinics.

**Trial registration:**

This work served as part of formative data collection for National Institute of Mental Health (NIMH) Trail NCT03711786 registered on 10th October, 2018.

## Background

Globally, depression is a leading cause of morbidity and mortality [1–3]. In low and middle-income countries (LMICs), mental health disorders account for almost the same disease burden as HIV/AIDS, 11.1 and 13% respectively [4, 5]. LMICs in general, and Malawi in particular are undergoing epidemiologic transitions away from infectious diseases and towards chronic non-communicable diseases (NCDs) [6–8]. Co-occurrence is frequent due to the high burden of depression and NCDs, and is associated with worse NCD outcomes [9].

Due to the complexities of co-occurrence, depression interventions alone have not been effective at improving NCD outcomes, increasing calls for integrated services [10–13]. As NCD care systems in LMICs expand, integrated services may facilitate successful treatment of patients with co-occurring NCDs and depression [14–16]. Integrated services are new to Malawi, and a lack of understanding on how to implement such services may stymie improvements in clinical outcomes. Therefore, clarifying patients’ attitudes towards depression in general, and the implementation of integrated services in particular, is required.

This qualitative study served as formative work for a trial of implementation strategies to identify effective means of integrating depression services (i.e., screening, psychosocial counseling, and anti-depressant medication management) into NCD services in Malawi. To guide this effort, we conducted in-depth qualitative interviews with NCD patients to answer the research question: what are Malawian NCD patient attitudes towards depression, and integrated care?

## Methods

### Sampling

Ten District Hospitals in Malawi’s Central Region were selected based on proximity to a Lilongwe based research organization in partnership with this study. The partner organization assisted with study design, staffing, transportation, translation/transcription, analysis, and other logistical support. Nine of the District Hospitals represented rural/semi-urban areas of the Central Region with just one hospital serving patients from a urban area. While urbanicity was not an a priori focus of our sampling strategy, the proportion of rural-to-urban hospitals roughly mirrors the country’s population where 84% of Malawians reside in rural areas [17]. Twenty participants (2 patients from each hospital) were approached for in-depth interviews.

This study used a convenience sampling approach where NCD providers were asked to identify two patients as interview participants. This sampling strategy was necessary due to study budget limitations which allowed for the Lilongwe-based interviewers to make just one trip to each District Hospital and thus, one opportunity to interview patients present during their visit. Convenience sampling is common in qualitative research and the inferences made in this analysis apply only to patients in Malawian NCD clinics [18]. Patients were approached by research staff for informed consent and enrollment. This study was approved by both Malawian and American research ethics committees prior to implementation (NHSRC Approval #1925 granted 3rd April, 2018; UNC IRB Study #17–3110 approved on 28th March, 2018).

### Final sample

One-on-one interviews averaged 59 min each and were completed with 20 patients, 2 from each NCD clinic (Table 1). Participants varied the most by urbanicity, however, this reflects Malawi’s largely rural population. Gender and age were rather evenly distributed among the participants, and most reported receiving either primary or secondary education. Our analysis did not find variation by any demographic characteristics presented in Table 1. All participants approached for enrollment consented, enrolled, and completed their interviews. The authors felt that saturation had been reached at the end of these 20 interviews and thus, participants were interviewed only once.

**Table 1:** Participant Sample

	Patients
Gender	
Female	11
Male	9
Age	
25–40	5
41–50	5
51–60	6
61–70	4
Rurality	
Urban	2
Rural	18
Education	
None	1
Primary	6
Secondary	10
Tertiary	3

### Data collection

A semi-structured interview guide was developed in partnership with local implementing partners. The interview guide first asked for patients’ reactions to a series of vignettes rather than asking patients directly about their own experiences with depression. The vignettes depicted individuals with varying levels of depression symptoms and asked patients about their attitudes regarding each individuals’ symptoms, the causes of their symptoms, and treatments. Patients were then asked about their own experiences with depression. Finally, the interviewers provided a description of psychosocial counseling and antidepressant medication services, both of which are relatively novel treatments in Malawi at the time of this study and asked about the appropriateness of such services. Appropriateness in this context is defined as the perceived fit, relevance, or compatibility of depression screening and treatment in Malawian NCD clinics [19].

The patient interview guide was written in English and translated into Chichewa.

Two female Malawian post-secondary research assistants fluent in Chichewa and English completed qualitative data collection training and interviewed participants from June – August 2018 (authors AT and MM). The data collectors had no prior relationship to the participants and, as part of the informed consent process, introduced the aims of the study prior to beginning each interview. All interviews were conducted in private rooms within the NCD clinics and audio recorded. Interviews conducted in Chichewa were translated and transcribed into English in one-step. The study team was not able to return transcripts to participants for comment/correction due to logistical constraints. Participants did not speak English, and a subset did not read or write. Because the audio-recordings were translated from Chichewa into English in one-step, a verbatim review of the English transcript (for reading participants) would require back-translation to Chichewa, an issue that one-step translation was designed to avoid. However, all interviews were checked for quality of transcription by a supervisor (author CZ). Further information of the ramifications, and supports in place to mitigate this method, are discussed further in the limitations section.

### Analysis

All transcripts were first read to gain familiarity with the data. During the second reading, the first author took notes for each interview and developed initial code categories and questions for the data collectors regarding language and transcription [20]. Through a process of concept-driven coding, the first author utilized their reading of the data, depression and implementation science literature, conversations with the research team, and their own experiences to create an initial codebook [21]. Initial code categories included depression causes, symptoms, treatments, and treatment sources. The first author used Dedoose v8.0.4 for coding and memo-writing, a key analytic tool. Code application categorized and reduced the raw data. Analytic memo-writing facilitated a practical approach that identified similarities, differences, and connections between codes [22]. Both processes were iterative, allowing for the addition of new codes and memo categories to emerge from the data. Coding matrices produced frequencies for various code categories. The full research team was engaged in ongoing conversation throughout the analysis in order to refine and validate findings.

## Results

The sections below describe: [1] patient perspectives on the causes, symptoms, and treatments for varying levels of depression [2]; the appropriateness of integrated services; and [3] the role of the patient-provider power dynamic.

### Patient perspectives on causes, symptoms, and treatments for depression

Patients agreed upon the causes and symptoms of depression, noting that it is often caused by interpersonal conflict (i.e., arguing with a family member) or worries regarding unemployment and financial strain. Regarding symptoms, patients consistently described depressed individuals as withdrawing from friends and family, developing exacerbated NCD symptoms, and experiencing suicidal ideation. The vast majority used social support (i.e., talking to a depressed individual about their problems to improve their mood) and prayer as helpful strategies for addressing depression, with prayer described as supplemental to social support. While 17 patients mentioned prayer at a Christian church, only 5 described it as a priority over other treatments. For instance, when asked how she would respond if experiencing moderate depressive symptoms, a hypertension patient noted:

I would go to church and pray even if it were for three days, I would definitely go for all the said three days so that whatever is in my heart, should come to an end (Female, age 35).

Social support itself was the most commonly reported treatment for moderate depression symptoms. For instance, a diabetes patient explained the process of treating depression through a supportive group of friends:

I am saying that these [depressed] people, mostly they don’t like to be open to their friends...Among [a group of friends], there can be someone who may have experienced the problem that you are going through and knows how to handle that problem. Such a person may help you to overcome the problem you are going through (Male, age 40).

A hypertension patient similarly discussed the use of social support to treat a man dealing with depression symptoms:

[A man dealing with depression] should be among other people so that they can chat with him and encourage him so that maybe he can stop the crying that he is doing. If they leave him alone, he can think of killing himself but they should be with him and assist him the way they can (Female, age 45).

In addition to social support and prayer, 17 patients described the hospital as a source of depression treatment, but only for individuals with severe depression and significant functional impairment. Less than half described medical intervention for treating moderate depression, suggesting the belief that medical intervention is more appropriate in cases of severe depression.

### Appropriateness of receiving depression services at an NCD Clinic

Although patients initially stated preferences for social support or prayer to treat depression, when asked about the integration of depression services within NCD clinics, every patient described integration as appropriate.

During the interview with the hypertension patient that prioritized prayer as a depression treatment, the interviewer discussed psychosocial counseling and asked the patient’s views regarding counseling at their NCD clinic:

It would be helpful because they will be able to disclose all their problems, if that specific person would guide and counsel him on what to do (Female, age 35).

When the interviewer described screening and medication, and similarly asked for the patient’s thoughts:

The medicine would be effective since they will be examined to reach a diagnosis. The reason why people are told to go to a health facility is for them to be examined so that a diagnosis can be ascertained. So it would be nice if that can be endorsed, if medicine would be made available (Female, age 35).

Here, the patient stated their initial preference for depression treatment as prayer at a Christian church. Once the interviewer described screening and treatment as a *medical process delivered through the NCD clinic*, the patient responded that the services would be appropriate.

After describing the integration of depression screening, psychosocial counseling, and anti-depressant medication within the NCD clinic, other participants who prioritized prayer as a treatment for depression symptoms similarly described appropriateness for integrated services. Initially stating:

It’s very difficult to deal with [depression symptoms] because you can go and associate with friends, but when you get back home you’ll still meet the problems. The most important thing that you can do is just to pray (Female, age 60).

And later reacting after the interviewer discussed depression medication at their NCD clinic:

When you are thinking a lot on something, that’s when you get depression. If there can be some medications for that that’s okay. We can all accept it (Female, age 60).

We noted a similar trend in patients who valued social support as a primary means of treating moderate depression. When the interviewer introduced depression screening for co-morbid patients at the NCD clinic, the same diabetes patient noted:

They would feel very happy because they would know that there is a way to diagnose their problem and get treatment. You know when a problem is diagnosed, you know there is treatment. So, diagnosis is very important (Male, age 40).

Similar to patients who prioritized prayer in treating depression symptoms, the patients who prioritized social support, or some combination of the two, all described the integration of screening and treatment at their NCD clinic as appropriate.

While all participants described depression service integration as appropriate, several expressed conflicting feelings regarding the effectiveness of anti-depressant medication. One patient first noted their preference for prayer and voiced skepticism regarding medication:

[Treating depression with medication] is not possible … because depression is something that you create through things that have happened to you, so it is up to you to accept and deal with (Female, age 43).

However, when asked what she would do if she had depression symptoms herself, the patient noted a preference for medical intervention in the form of screening:

[I would be] coming to the hospital so that the doctor should test me as if they test sugar or BP … They can identify that the illness is there because of the brain so they tell you what to do so you stop thinking too much (Female, age 43).

Despite the patient’s skepticism regarding medication, they were open to seeking depression care from their NCD provider. This openness reflects a flexibility in NCD patient attitudes given every patient described depression services as appropriate, despite initially voicing a preference for other treatment options. This flexibility appears grounded in a patient-provider power dynamic, rooted in patients’ respect for providers’ medical knowledge.

### Respecting medical knowledge – The patient-provider power dynamic and the importance of communication

When the interviewers explained the process of screening and treatment *at the NCD clinic*, depression became a topic of medical knowledge. Patients discussed a great respect for medical knowledge. For instance, a hypertension patient highlighted the difference between the same dietary recommendations offered by community members and a medical provider:

There is a difference, [community members] tell us that “do not eat this or do not eat this,” and we do not listen. But, when a doctor tells us that “if you eat this you are putting your life at risk,” we listen (Female, age 60).

Patients also related a respect for medical knowledge when given hypothetical scenarios described in depression vignettes. A hypertension patient described a possible interaction with her husband, pretending that he is the man in the vignette with depression:

He will understand the counselling from a health worker because he is afraid of the health workers. But for me to tell my husband about counseling, he will say, "What do you know!” but he is afraid of the health workers. “These people know something, they are well trained” and so he just listens attentively, and he may also just take medications (Female, age 59).

The notion of accepting medical recommendations out of fear introduces the issue of power. Describing a patient-provider interaction as charged with fear acknowledges the provider’s power to shape how a patient feels. A different patient expanded on this dynamic:

If you look at someone who is the health worker and is putting on that uniform it adds some grain of believability unlike someone that is from the community where we live. We believe that the health worker will answer most of our problems … The other reason why a health worker is more believable than someone from the community is because the health worker had to go to school to do what he or she does unlike member of the community whose education will always be questionable … The moment the patient sees the health worker, psychologically he or she feels they have been helped (Male, age 48).

The importance of medical credibility and power became more pronounced when interviewers asked how a patient might react when receiving a depression diagnosis. Given depression services would be new to NCD clinics, and moderate depression was not viewed as a medical condition, the ability to transfer medical knowledge to the patient becomes critical. Patients cautioned that the initial depression diagnosis could be jarring “because we have never thought of it before, that sadness can also be a disease.” The way in which a provider explains depression as a treatable medical condition, could potentially exacerbate mental health issues, or provide comfort and a clear path forward. A hypertension patient discussed the importance of patient education:

She can be stressed out. Upon hearing that she has been diagnosed with a medical condition she will feel bad. She will be thinking that the medical condition is permanent. There are two possibilities. The first one you may go to the hospital and the clinician will tell you “sorry mum we have found you with a wound. However, the good news is that the wound will get healed soon.” Or you may go to the hospital and be told that you have been diagnosed with a disease that will take almost forever. The second one could leave you stressed out (Female, age 49).

A diabetes patient described the same issue and added importance to the way a provider speaks to a patient.

For someone who has come from the village to understand the diagnosis, it will get him even more depressed because he will say “is being depressed even a disease?” It also depends on the person conducting the test. They can either make the person more depressed or they can help end the depression the person already has. Explain to them properly, in a slow, soft tone, tell them that you have been found with this disease and these are the causes, explain to them properly and counsel them (Male, age 52).

## Discussion

### Overview

The patients in our sample described how depression emanates most frequently from interpersonal conflict. When presented with vignettes of individuals with varying levels of depression, patients agreed that medical treatment was necessary for cases of severe depression. For moderate cases of depression, participants mentioned social support or prayer at a Christian church as common forms of treatment. However, when the interviewers described the process of depression screening and treatment with existing NCD services, all patients agreed that such services were appropriate. Despite agreeing upon the appropriateness of integrated services, patients expressed an existing gap in medical knowledge between themselves and medical providers. Some patients described how the introduction of depression screening and treatment could increase the gap, given that moderate cases of depression were not generally viewed as medical issues. Patients further expressed the importance of [1] the provider’s role in effectively educating them about depression and [2] communicating information about depression in a manner that would put them at ease, rather than exacerbate feelings of stress related to a depression diagnosis. These findings suggest the need for Malawian NCD providers to take caution in their interactions with patients regarding depression screening, diagnosis, education, and treatment.

The sections below describe: [1] the role of mental health literacy in shaping the patient-provider power dynamic; and [2] means of addressing the patient-provider power dynamic to improve clinical outcomes.

### The role of the mental health literacy gap

Participants viewed moderate depression as less of a medical issue, but recognized severe depression as requiring medical intervention. These findings align with other data from the region. In their review of mental health literacy publications from LMICs, Ganasen et al. (2008), describe several studies that found participants in LMICs perceived less severe mental illness symptoms as part of normal life experience, resulting in “talking it out” as a common treatment [23]. This idea echoes through our data given the vast majority of participants who described social support as a means of treating depression symptoms. While education on certain health issues like HIV transmission, risk factors, and treatments have been the subject of many peer education, mass media, and school-based interventions in Sub-Saharan Africa, community education efforts regarding common mental illnesses have received far less attention [23–26]. Ganasen et al. (2008) further note that improving mental health literacy among primary health care professionals is imperative to improving mental health literacy in patients [23]. We share this recommendation as well. Our patient data show similarly limited degrees of recognition for depression as a medical condition. As providers receive training in evidence-based depression screening and treatment, the gap in patient-provider medical knowledge may become further pronounced given the dearth of public education regarding mental illness, potentially exacerbating the existing power dynamic. Training providers to not only understand and recognize cases of depression, but also to effectively educate patients on the risk factors, causes, symptoms, and treatments are key to influencing how patients feel about diagnosis and treatment.

### Addressing the patient-provider power dynamic

While the unequal distribution of knowledge in medical settings and the patient-provider power dynamic are somewhat universal, our findings further contextualize these phenomena within the Malawian NCD clinical setting [27, 28]. Participants described how providers can heavily shape patients’ feelings about depression, suggesting the interaction must be handled with care. In their review of World Health Organization (WHO/UNAIDS HIV) policy guidelines, Evans & Ndirangu (2009) noted that patient-provider relationships in hierarchical medical systems give way to a power dynamic where patients feel unable to go against their provider’s advice [29]. Relating their findings to depression services, an unintended consequence of offering depression screening and treatment as part of regular NCD services may be that patients agree to screening without fully understanding what the screening is for.

Aside from providing adequate depression education, the *manner in which a provider speaks to a patient* was described as critical by the patients in our sample. Current recommendations for treating depression in primary care include patient engagement and education, close follow-up, and a commitment to adjusting treatments or consulting with mental health specialists until depression is improved [30]. Evidence also suggests a better patient-provider relationship is associated with a higher quality of depression care, as well as improved depression and NCD outcomes [31–33].

Current depression treatment guidelines call for providers to closely monitor patients and their responses to depression treatments [34]. However, in their review of good communication in psychiatric practice, Priebe et al. (2010) note that while most general medical training includes patient communication, more nuanced and careful communication is required for successful patient-provider relationships in psychiatric settings [35]. Current guidelines also describe identifying markers of response at the earliest stages of treatment as a critical challenge [34]. However, a patient experiencing complications with treatment, in an unbalanced system of power, may fear that communicating such complications challenge their provider’s medical knowledge. Strategies are required to mitigate this issue in treating depression in Malawi. While the patient-provider relationship intervention literature is underdeveloped in LMICs, interventions from HICs that focus on increased patient education, longer and more frequent visits, and increased encouragement regarding medication adherence have shown significant increases in anti-depressant adherence [36]. Patients of a provider who gives clear instructions about the treatment; who relates to patients in an approachable, or supportive manner; who solicits and listens to client’s views; and who uses a participatory manner of problem-solving are significantly and positively associated with patient knowledge and satisfaction with anti-depressants [37]. The need to train NCD providers and equip them with strategies to mitigate their position of power is key to the successful integration of evidence-based depression services in Malawian NCD clinics.

### Strengths and limitations

Our study’s strengths include exploration of a novel research area, and a sample of 10 clinics reflecting heterogeneity in Malawian clinical care. Despite the growing risk of NCDs in sub-Saharan Africa, and calls to increase integration of depression and NCD services in LMICs - to our best knowledge, no other qualitative literature exists on Malawian NCD patients’ attitudes and beliefs regarding depression and its integration with NCD services [6–8, 38, 39]. We hope that addressing this gap may lead to improvements in the roll out of integrated services in Malawi. Our analysis also allowed us to reach saturation on several themes across a sample that included 10 hospitals, representing a large geographic area and clinic level heterogeneity (e.g., variation in rurality, staff size, and caseloads). Representativeness and generalizability are rarely the focus of sampling in qualitative research and were not an a priori focus of our sampling strategy. Despite this, we believe that the saturation derived from our analysis, across a diverse clinic and patient population, hold relevance as integration expands across the country.

Our findings should also be understood within the context of several limitations. First, participants were interviewed in Chichewa but recordings were translated and transcribed to English in one step due to resource limitations. Further, in an effort to accelerate data preparation, about half of the transcription was completed by research assistants that played no role in data collection. Challenges inherent in translation alone are well documented in qualitative inquiry and both the one-step transcription and multiple transcribers likely take away from the quality of these data [40]. In order to mitigate this, the data collectors and their supervisor listened to each recording and matched them with each translated transcript to ensure accuracy. Additionally, interviews were carried out within district hospitals, which may have influenced patients to discuss depression treatment in a medical setting more positively. Traditional means of treatment for mental health are relatively common in Malawi and were rarely found in our data [41]. However, patients seek traditional based treatment for many conditions and researchers have consistently called for increasing integrative treatment [42–45]. Similar to our recommendations for provider sensitivity to patients’ beliefs, these considerations should remain inclusive of patients’ traditional or other spiritual beliefs related to depression.

## Conclusion

This study fills a gap in the literature regarding Malawian NCD patients’ perspectives regarding depression and integrated depression services. As calls for integrated services escalate in Malawi, an understanding of patients’ perspectives can serve to improve the appropriateness of implementation. Our data show that attitudes regarding the treatments for depression varied mostly between preferences for prayer and social support. Additionally, patients believed depression warranted medical treatment for only severe cases. Despite various treatment preferences and the fact that moderate depression was rarely viewed as a medical issue, patients described depression services in NCD settings as appropriate. The patient-provider relationship was discussed as central to a patient’s experience with depression screening and treatment. Patients described how medical knowledge is highly regarded and how providers wield the power to shape patients’ reactions to depression diagnoses, underscoring the importance of provider communication around a depression diagnosis and treatment.

In order for Malawian NCD providers to adequately educate, screen, and treat patients for depression, they will need to address this power imbalance through the use of strategies designed to mitigate their position, and engage patients to form a more collaborative treatment relationship.

## Data Availability

The datasets generated and/or analyzed during the current study are not publicly available given their focus on small scale program specific outcomes but are available from the corresponding author on reasonable request.

## Abbreviations

LMICs: Low and middle-income countries
NCDs: Non-communicable diseases
NIMH: National institute of mental health
HICs: High-income countries
WHO: World Health Organization
